# Seasonal Changes in the Biochemical Composition of Dominant Macroalgal Species along the Egyptian Red Sea Shore

**DOI:** 10.3390/biology12030411

**Published:** 2023-03-07

**Authors:** Marwa Kamal, Neveen Abdel-Raouf, Khairiah Alwutayd, Hamada AbdElgawad, Mohamed Sayed Abdelhameed, Ola Hammouda, Khaled N. M. Elsayed

**Affiliations:** 1 Botany and Microbiology Department, Faculty of Science, Beni-Suef University, Beni-Suef 62521, Egypt; 2 Department of Biology, College of Science and Humanities in Al-Kharj, Prince Sattam Bin Abdulaziz University, Al-Kharj 11942, Saudi Arabia; 3 Department of Biology, College of Science, Princess Nourah bint Abdulrahman University, P.O. Box 84428, Riyadh 11671, Saudi Arabia; 4 Integrated Molecular Plant Physiology Research, Department of Biology, University of Antwerp, 2020 Antwerp, Belgium

**Keywords:** Red Sea, macroalgae, seasonality, molecular identification, biochemical composition

## Abstract

**Simple Summary:**

Macroalgae play a significant role as primary producers in marine ecosystems. The most dominant species at the three collection sites studied along the Egyptian Red Sea were seasonally harvested. Among several species collected, five dominant macroalgae (*Caulerpa prolifera*, *Acanthophora spicifera, Cystoseira myrica*, *Cystoseira trinodis* and *Turbinaria ornata*) were selected for further studies. These macroalgae were identified using morphological and molecular characteristics. During summer and winter, the mineral content and biochemical composition of the selected macroalgal species were evaluated. These analyses indicated that macroalgae are rich in minerals as well as primary and secondary metabolites. Moreover, the findings reported that the macroalgae studied possess high nutritional value in the summer more than in the winter season.

**Abstract:**

Macroalgae are significant biological resources in coastal marine ecosystems. Seasonality influences macroalgae biochemical characteristics, which consequentially affect their ecological and economic values. Here, macroalgae were surveyed from summer 2017 to spring 2018 at three sites at 7 km (south) from El Qusier, 52 km (north) from Marsa Alam and 70 km (south) from Safaga along the Red Sea coast, Egypt. Across all the macroalgae collected, *Caulerpa prolifera* (green macroalgae), *Acanthophora spicifera* (red macroalgae) and *Cystoseira myrica*, *Cystoseira trinodis* and *Turbinaria ornata* (brown macroalgae) were the most dominant macroalgal species. These macroalgae were identified at morphological and molecular (18s rRNA) levels. Then, the seasonal variations in macroalgal minerals and biochemical composition were quantified to determine the apt period for harvesting based on the nutritional requirements for commercial utilizations. The chemical composition of macroalgae proved the species and seasonal variation. For instance, minerals were more accumulated in macroalgae *C. prolifera, A. spicifera* and *T. ornata* in the winter season, but they were accumulated in both *C. myrica* and *C. trinodis* in the summer season. Total sugars, amino acids, fatty acids and phenolic contents were higher in the summer season. Accordingly, macroalgae collected during the summer can be used as food and animal feed. Overall, we suggest the harvesting of macroalgae for different nutrients and metabolites in the respective seasons.

## 1. Introduction

The Red Sea is known to be the northernmost tropical sea in the world, possessing a remarkable geography [1]. It is considered a landlocked and largely unperturbed marine ecosystem, which is situated in one of the world’s hottest places along a small basin separating the continents of Asia and Africa [2]. Its coastal areas of Egypt are very interesting to many researchers [3]. This is because the coastal areas of the Red Sea possess more biotope and species diversity than the Mediterranean Sea and the world’s oceans [4]. The Red Sea ecosystem comprises macroalgae, mangroves and coral reefs [1]. Macroalgae diversity performs an important ecological role through the cycling of carbon, nitrogen and phosphorus, which results in the regulation of marine water quality [5].

Based on their pigmentation, morphology, anatomy and biochemical composition [3], macroalgae are classified into three categories: red (Rhodophyta), brown (Phaeophyta or Ochrophyta) and green (Chlorophyta) [6]. Each class of macroalgae is characterized by particular kinds of pigments, which give them their definite colors as well as distinctive group names [7]. Globally, more than 4000 species of Rhodophyta, 1500 species of Phaeophyta and 900 species of Chlorophyta have been recorded [8]. Approximately 500 species of macroalgae were listed in the Red Sea [9]. Recently, the macroalgal biomass in the Red Sea recorded an apparent increase, which may be attributed to nutrient enrichment from urban and aquaculture outflow, as well as reduction in herbivores [2]. It is well known that the surrounding environment can influence the biodiversity and abundance of macroalgal flora, allowing some species to predominate over others [2,10].

Seaweeds are marine macroalgae that inhabit the littoral zone [11]. Seaweeds are characterized as non-vascular plants, which represent the primary producers in oceans and belong to the Protista not Planta kingdom [3]. They grow from intertidal to shallow coastal waters, in addition to deep waters, up to 180 m in depth [12]. They can provide oxygen, food resources and shelter substrates for many aquatic organisms. The floristic composition of marine macroalgae [13], in addition to their distribution and periodicity sequence, can be used for estimating several ecological changes [14]. For example, they help in reducing ocean acidity and offer a solution to global warming [15,16]. Moreover, they support the diversity and productivity of some communities because they provide oxygen, food, as well as habitat for many kinds of aquatic biota [12].

Seaweeds attract attention as one of the most biologically active resources in nature due to their great content of bioactive compounds. Macroalgae are known to be a wealthy source of dietary fiber, essential amino acids, nutrients, vitamins, antioxidants and lipids [3,17]. Thus, they are valuable natural sources for fertilizers and plant growth regulators, food commodities, animal feeds and perform a crucial role in agriculture and horticulture [18,19]. Recently, seaweeds have also been used for a variety of purposes, including health benefits, biofuel production, cosmetics, pharmaceuticals, textiles and bioplastic packaging [20,21]. In this context, seaweeds rich in bioactive components [12], including antioxidant, anti-pigmentation, anticancer, anti-wrinkling and antimicrobial activities, have been of particular interest [7,22,23].

The quality and concentration of bioactive compounds of seaweeds depend on various factors, including the season, geographic location, harvesting period, in addition to biotic factors, such as herbivory or direct competition with other organisms, and abiotic factors, such as salinity, temperature, pH and nutrient composition of water [24,25]. These factors could stimulate or inhibit the production of macroalgal bioactive constituents [26]. The ability of macroalgae to produce distinctive secondary metabolites, such as polysaccharides, proteins, lipids and phenolic compounds, enables them to quickly adapt to changes in the marine environment, including temperature and solar radiation [27]. Moreover, the great content of these metabolites in seaweeds may differ significantly according to the taxonomic group, geographical, seasonal and physiological variations [28,29]. The formation of marine macroalgal communities is regulated by a set of restrictions, such as light, depth, temperature and nutritional content. As a result of macroalgal species’ diversity and availability being affected, the marine environment ultimately changes [30]. In the Red Sea, macroalgae are known to be one of the most significant biological resources in coastal marine ecosystems, as well as supporting some communities’ diversity and productivity because of their important role as primary producers in the marine environment [31].

Seaweed communities are considered significant as an indicator of environmental stress, as their distribution and abundance are affected by disturbances, such as desiccation, high temperatures and competition with coastal flora and fauna [32]. Therefore, it is very important to study their variations and distribution at different times and places [33]. In this study, green, red and brown macroalgae species were collected from the Red Sea shore, Egypt, during four seasons. Out of several species collected, five species were selected based on their dominance throughout the four seasons of the year in the geographical locations under investigation. These five selected seaweeds were identified based on morphological and molecular characterization. Then, the biochemical compositions of the selected seaweeds, including primary metabolites (carbohydrates, amino acids (AAs), fatty acids (FAs) and organic acids), secondary metabolites (phenolics) and mineral profiles, were analyzed to evaluate the influence of seasons, i.e., summer and winter. To our knowledge, the present study is the first to evaluate the seasonal impact on the biochemical compositions of macroalgal species. This was also required in order to determine their potential use in human food and other industries.

## 2. Materials and Methods

### 2.1. Collection Sites, Seasonal Climate Conditions and Identification of Macroalgae

**Figure 1:**
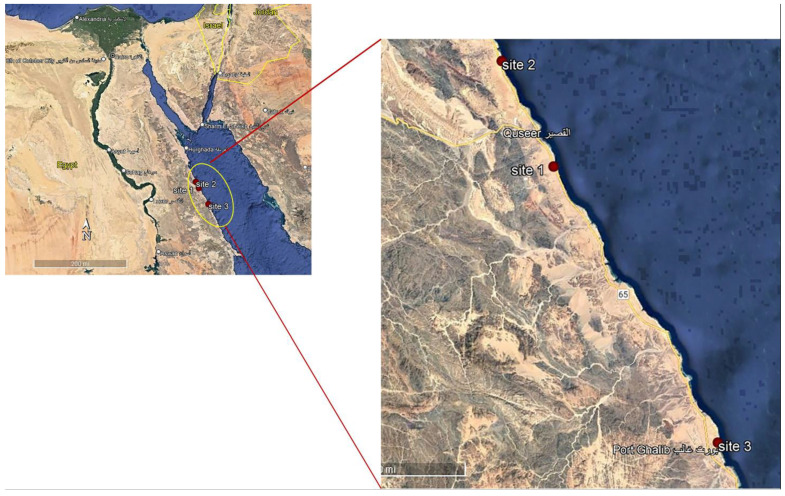
Map illustrating the three collection sites along the Red Sea, Egypt.

Macroalgal specimens were collected from three sites at 7 km (south) from El Quseir (26°2′34.02″ N; 34°18′51.51″ E), 52 km (north) from Marsa Alam (26°11′30.75″ N; 34°13′43.92″ E) and 70 km (south) from Safaga (25°32′56.35″ N; 34°38′16.88″ E) along the Red Sea coast, Egypt, seasonally, from summer 2017 to spring 2018 during low tides when seaweeds are exposed (Figure 1). These sites were selected because (1) they are fertile seacoasts and they are markedly rich in flora and fauna, and (2) there is an absence of industrial activities, as well as (3) a significantly lower population of habitants. The quadrate technique (steel quadrate 100 × 100 cm) was applied for the collection of macroalgal samples from the three collection sites [34]. Five quadrate samples were collected at each site. Macroalgae were harvested at their maturation stage manually and washed thoroughly in sea water to remove potential contaminants, such as adhering impurities, sand particles, rock debris, epiphytes and animal castings. The fresh biomass was collected in polyethylene bags containing sea water to prevent evaporation and washed with tap water followed by distilled water to remove excess salts. The dried samples were fine-powdered using a food mixer and stored in labeled plastic bags for further use [35]. Some of the collected seaweeds were preserved for identification. The relative abundance of each macroalgal species was determined according to the following equation: Abundance % = No of individuals of a given species × 100 ÷ Total no. of all species [36]. The climate conditions of these sites were as follows. Water temperature varied between 15.8 and 18.5 °C in the winter months and 31.7 and 32.7 °C in the summer at day time at the selected sites. During the summer, the pH values were slightly alkaline; they fluctuated between 7.72 during the winter at Site 3 and 7.89 at Site 1 (Table 1). At first, the macroalgal samples collected were identified based on their morphological characteristics with taxonomic references [37]. The morphological identification was followed by molecular identification.

### 2.2. Molecular Identification

DNA was isolated using the Cetyl Trimethyl Ammonium Bromide (CTAB) method from approximately 400 mg of macroalgal powder ground in liquid nitrogen [38]. The purity and concentration of extracted DNA were determined using a spectrophotometer at 260 nm and 280 nm. Purity was measured at the ratio of A 260: A 280 using agarose gel electrophoresis. The purified DNA isolate was amplified through the polymerase chain reaction (PCR) process using 18S rRNA primers (Table 2). Basic local alignment search tool (BLAST) analysis was used to determine similarities in GenBank to confirm the species of the macroalgal samples collected. The National Center for Biotechnology NCBI (blast.ncbi.nih.nlm.gov) was used to carry out this analysis by entering the complete sample sequences into the BLAST analysis. Phylogenetic trees were constructed using the MEGA X program.

### 2.3. Physico-Chemical Analysis of Water Samples

Samples of water (approx. 2 L) were collected from the study sites in clean, plastic bottles and transferred to the laboratory in cold condition. Water temperature and pH were measured in situ using Hydrolab, Model (Multi Set 430i WTW). For the other chemical analysis, water samples were collected and transferred to the laboratory to measure the chemical parameters. Calcium (Ca^++^), magnesium (Mg^++^), salinity, total hardness as CaCO_3_, chloride (Cl^−^), Sulfate (SO_4_^−^), bicarbonate (HCO_3_^−^), nitrate (NO_3_^−^), total phosphate (TP), copper, zinc and lead were measured following the protocol of the American Public Health Association standard methods (APHA) [39].

### 2.4. Primary Metabolites’ Analysis

The sugars, amino acids (AAs) and fatty acids (FAs) and contents of macroalgal biomass were evaluated and recorded in both seasons (summer and winter). Sugars were measured in an acetonitrile/water (2 mL, 1:1, *v*/*v*) extract and determined using high-performance liquid chromatography (HPLC) according to Alasalvar et al. [40]. Individual sugars were measured using standard curves built using definite concentrations of standard sugar solutions from 1 to 10 mg/100 mL of acetonitrile/water (1:1, *v*/*v*).

The AAs of macroalgal samples were measured according to Sinha et al. [41] using 1 mL of 80% (*v*/*v*) aqueous ethanolic extract. Seaweed extracts were centrifuged, and then, the supernatant was evaporated under vacuum. Pellets were dissolved in 1 mL of chloroform, and the suspension was re-extracted using 1 mL of HPLC-grade water. Then, the aqueous phase was gathered after centrifugation and filtered using 0.2 μM Millipore microfilters. AAs were analyzed using a Waters Acquity UPLC-tqd system (Milford, Worcester County, MA, USA) equipped with BEH amide 2.1 × 50 columns.

The FAs of macroalgal samples were estimated using GC/MS using aqueous methanolic extract (1:1 *w*/*v*) until discoloration occurred according to Torras-Claveria et al. [42]. The FAs of macroalgal extracts were identified with GC/MS using a Hewlett Packard 6890, MSD 5975 mass spectrometer (Hewlett Packard, Palo Alto, CA, USA). Different FAs were quantified with the NIST 05 database and plant-specific databases.

The organic acids of macroalgal samples were measured according to De Sousa et al. [43]. Samples of macroalgae powder were milled and extracted using 0.1% phosphoric acid containing butylated hydroxyanisole. The internal standard (ribitol) was added during the extraction steps. After centrifugation for 30 min at 14,000 rpm, the supernatant was transferred to new tubes for HPLC evaluation (LaChrom L-7455 diode array, LaChrom, Tokyo, Japan). Methanol was used for samples’ elution as mobile phase A and 5% potassium dihydrogen phosphate (pH 2.5) as mobile phase B at 0.5 mL/min and 40 μL injection volume.

### 2.5. Minerals’ Analysis

Macroalgal samples were digested using HNO3/H2O (5:1 ratio) in an oven. Various minerals were measured using mass spectrometry (ICP—MS Finnigan Element XR; Scientific, Bremen, Germany) according to Ref. [44]. Standard mixtures were prepared in 1% nitric acid.

### 2.6. Phenolic Compounds

Total polyphenols and flavonoids were assessed in macroalgal biomass extracted in 80% ethanol. The phenolic content was quantified by the Folin–Ciocalteu method [45], while flavonoids were determined by the modified aluminum chloride colorimetric method [46]. Tocopherols were determined using hexane extract quantified by HPLC according to Siebert et al. [47].

### 2.7. Statistical Analysis

The results were expressed as mean ± SD (standard deviation) and analyzed by one-way ANOVA using IBM SPSS Statistical software package (SPSS^®^ Inc., Chicago, IL, USA). In cases of significant interactions between the factors, one-way ANOVA was performed for each factor, and Tukey’s multiple range tests were used to determine significant differences among means between the two seasons of the same species (*p* < 0.05). A significance level of *p* < 0.05 was used for rejection of the null hypothesis. All experiments were carried out in three replicates (*n* = 3).

## 3. Results and Discussion

### 3.1. Macroalgal Species Collection

**Figure 2:**
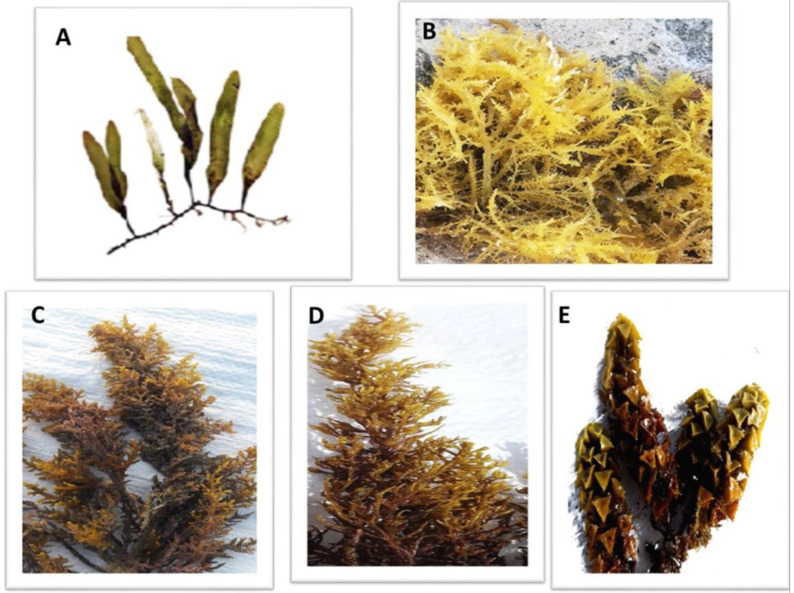
The dominant macroalgal species collected from three sites along the Red Sea shore, Egypt. (**A**) *Caulerpa prolifera*, (**B**) *A. spicifera*, (**C**) *Cystoseira myrica*, (**D**) *Cystoseira trinodis* and (**E**) *T. ornata*.

The dominance of macroalgal species along the Red Sea coast was determined according to the relative abundance of species from all collection sites throughout the year [36]. The green macroalgae (*C. prolifera*), the red macroalgae (*A. spicifera*) and the brown macroalgae (*C. myrica, C. trinodis* and *T. ornata*) (Figure 2) were the most prominent macroalgal species among all collected macroalgae.

**Figure 3:**
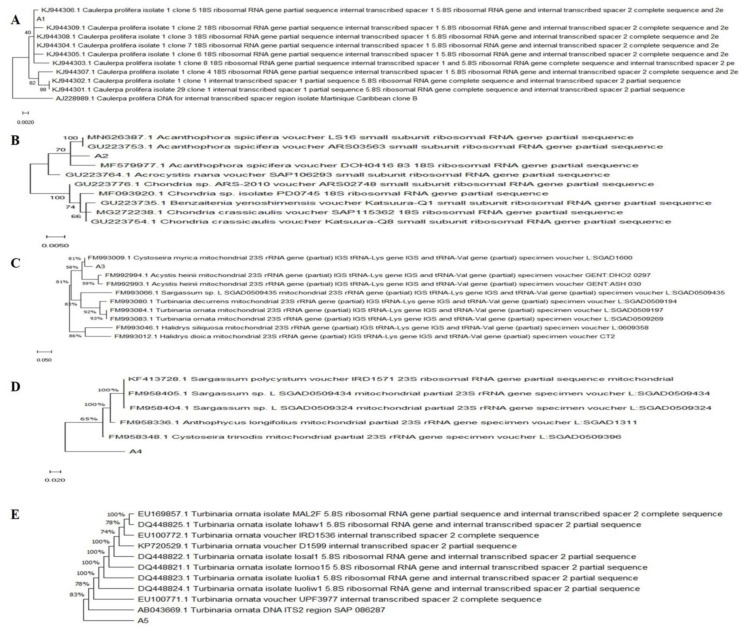
Phylogenetic tree of 18s rRNA sequences of macroalgae. (**A**) *Caulerpa prolifera*, (**B**) *A. spicifera,* (**C**) *Cystoseira myrica*, (**D**) *Cystoseira trinodis* and (**E**) *T. ornata.* constructed by NCBI/BLAST.

For years, the biodiversity of these seaweeds has been largely classified based on their morphological features [48]. Recent developments have inspired scientists to use molecular approaches to investigate the biodiversity of marine macroalgae [49]. Molecular studies were used by algal taxonomists for species’ discovery and identification, in addition to many routine taxonomic studies [50]. Therefore, the five dominant seaweeds collected were first morphologically identified, followed by molecular identification using 18S rRNA sequencing. The sequences of 18S rRNA were analyzed on NCBI using the BLAST tool to determine the sequences’ percentage of similarity with the sequences in GenBank. All of the obtained sequences corresponded to known macroalgal species with significant sequence similarity. Based on the results of phylogenetic tree analysis, the harvested macroalgal species were closely related to *Caulerpa prolifera*, red seaweed *Acanthophora spicifera*, brown seaweeds *Cystoseira myrica*, *C. trinodis* and *Turbinaria ornata*, respectively (Figure 3).

### 3.2. Minerals’ Level Change with Season and Species

Macroalgae accumulate minerals, which are necessary for seaweeds survival, as well as improve their nutritional value and as a medicinal source [32,51]. Fifty-two essential minerals, including macrominerals, such as Na, K, Ca, Mg and P, and trace elements, such as Cd, Fe, Zn, Cu and Mn, were identified. The minerals’ profiles of the five macroalgae investigated in this study exhibited various amounts of essential metals. P, K, Na and Mg were the most abundant elements among the different species. Their concentrations were as the the following ranges: 1.65–5.62 mg/g dry weight (DW), 0.64–2.54 mg/g DW, 0.29–0.91 mg/g DW, and 0.23–0.82 mg/g DW, 0.64–2.54 mg, 0.29–0.91 mg, and 0.23–0.82 mg, respectively. Significant difference at *p* < 0.05 was observed between the content of minerals in the summer and winter. *C. prolifera* and *T. ornata* had a high content of K in the winter season, but high content of K was recorded in *A. spicifera* and *C. myrica* in the summer. *C. trinodis*, *C. prolifera*, *A. spicifera* and *T. ornata* had a high content of P in the winter, but *C. myrica* and *C. trinodis* had a high P content in the summer. Na and Mg rendered the same results with different tested macroalgae. Significant increase was observed in the winter season for *C. prolifera*, *A. spicifera* and *T. ornata*. In contrast, significant increase was observed in the summer season for both *C. myrica* and *C. trinodis* (Table 3).

Macroalgae do not biosynthesize minerals, but they absorb them from the surrounding environment based on many factors, such as temperature, pH, salinity and light [52]. Thus, both internal and external factors have an impact on minerals’ accumulation in macroalgae. The former involve sulfhydryl ester, amino, carboxyl, hydroxyl, proteins and/or lipids, while the latter include sea water, temperature, salinity, pH and disruptions [53]. Regarding their biological and nutritional value, Ca is a crucial element in the body skeleton, in heart strength and smooth muscle contraction, in addition to the nervous and muscular equilibria [54], while Mg is a very important cofactor of several enzymes, including those involved in respiration. Other minerals, such as Fe, Mg, Cu, Zn and Co, are involved in several metabolic processes, as well as working as enzyme cofactors [32]. According to metal analysis of the four seaweeds *Laminaria digitata*, *L*. *hyperborea*, *Saccharina latissima* and *Alaria esculenta*, the concentrations of K and Na in the winter were more than the doubleof their concentrations in the summer [55]. The current result is in line with previous findings for *Laminaria digitata* [56]. Saldarriaga-Hernandez et al. [57] reported a high concentration of P in *Sargassum* and concluded that *Sargassum* is recommended as an alternative source of P. A similar result was described by Gaillande et al. [58] who indicated that high quantities of Na, K, Ca and Mg were also reported in *Caulerpa* species.

### 3.3. Species and Seasonal Variation in Primary and Secondary Metabolites

In this study, seasonal variations in macroalgal biochemical composition were observed, which affect the apt period for harvesting based on the nutritional requirements for commercial utilizations. Seasonal characterization is a prerequisite for future valorization of macroalgal biomass as a component of feed additives or fertilizers [51]. Thus, it is important to understand these variations in the production of biologically active compounds in order to determine the ideal time for harvesting macroalgal biomass based on its proposed applications in food, animal feed, biofuel production, pharmaceutical and various industries.

Several studies proved the effect of seasonality on the biochemical constituent of different species of seaweeds. Kumar et al. [59] observed significant individual differences in the biochemical composition of all investigated marine macroalgae. Ajayan et al. [60] studied the fatty acid contents, metals and other elemental compositions of 25 macroalgal species and proved that the lipids, proteins and carbohydrate levels varied significantly among the species studied. For instance, Samanta et al. [61] illustrated that the chemical composition of *Agarophyton vermiculophyllum* was changed by variable climatic conditions, such as temperature, pH and nutrient availability. Pérez et al. [62] proved that seaweed harvested in the summer showed superior physiological activities as a result of the presence of active metabolites, such as fatty acids, pigments, phlorotannins, lectins, terpenoids, alkaloids and halogenated compounds, as a pattern of adaptation. Overall, more studies are required to evaluate the use of macroalgae as a healthy and sustainable alternative in the nutraceutical, cosmetics, as well as well-being industries because seaweed exploitation in Egypt is still in its early stages [63].

#### 3.3.1. Carbohydrates

**Figure 4:**
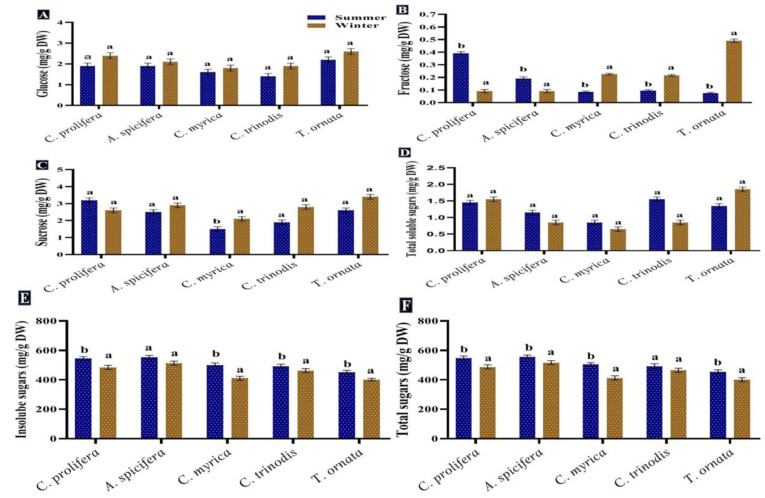
Seasonal variations in the concentrations (mg/g DW) of (**A**) Glucose, (**B**) Fructose, (**C**) Sucrose, (**D**) Soluble sugars, (**E**) Insoluble sugars and (**F**) Total sugars in macroalgae *Caulerpa prolifera, A. spicifera, Cystoseira myrica, Cystoseira trinodis*, and *T. ornata*. Values are shown as means ± S.E. (*n* = 3). Different letters show significance between the two seasons of the same species (*p* < 0.05).

Carbohydrates are considered the primary source of energy in the majority of human diets in addition to their importance in respiration and other metabolic processes [64,65]. Furthermore, they can be used for biofuel production [66]. In the present study, total sugars recorded a high content in the summer season for all macroalgal species tested. Red macroalgae *A. spicifera* had the greatest concentration (564.7 mg/g DW), followed by green macroalgae *C. prolifera* (557.9 mg/g DW), while brown macroalgae showed minimum contents (Figure 4). Monosaccharide glucose exhibited a different pattern, whereby a significant increase in glucose quantity was observed in the winter season in all macroalgae tested. The highest content of fructose was recorded in the winter for brown macroalgae in contrast to red macroalgae *A. spicifera* and green macroalgae *C. prolifera*.

The result obtained revealed that the greatest content of total sugars was found in the summer season in all macroalgae investigated. A similar pattern was noticed by Khairy and El-Shafay [64] who reported that the highest amount of carbohydrates in *U. lactuca* and *P. capillacea* was produced during the summer season. According to García-Sanchez et al. [67], *Sargassum* exhibited rapid growth during the summer season owing to greater sunlight exposure, storing carbohydrates for the rainy season, which is characterized by reduced photosynthesis. Variations in carbohydrates’ production among macroalgal species may be attributed to their various life cycles and abiotic oscillations [65].

#### 3.3.2. Proteins and Amino Acids 

Proteins are macromolecules and serve a variety of functions in all living organisms, including repair and maintenance, mechanical support and energy [32]. A total of 19 AAs were evaluated in the five macroalgae, including essential (EAAs) (which must be obtained from food) and non-essential amino acids (NEAAs). Lysine, histidine, phenylalanine and valine were the most prominent EAA. Lysine concentration was somewhat higher in the summer season in *C. prolifera, A. spicifera* and *T. ornata*, unlike the concentrations in *C. myrica* and *C. trinodis*, which were higher in the winter season. The content of phenylalanine in the summer was double that in the winter (significant difference at *p* < 0.05). There was a non-significant difference at *p* < 0.05 in the histidine level between both seasons. Glycine, alanine, asparagine and glutamic acid were the most abundant NEAA. There was a significant difference at *p* < 0.05 in their levels between both seasons. Glycine content was the highestamong both EAAs and NEAAs. Glycine concentration was increased by 20–30% in the summer season for all seaweeds studied, except *C.trinodis*. The greatest levels of alanine were observed during the summer in *C. prolifera* and *T. ornata*, while they were higher in *A*. *spicifera, C. myrica* and *C. trinodis* during the winter season. *A. spicifera* and *C. trinodis* had a high content of asparagine (4.49 mg/g DW) in the summer, while *T. ornata* had a high content of asparagine (5.04 mg/g DW) in the winter (Table 4).

The quality of the protein is just as important as its quantity. The protein quality of foods is frequently assessed by the amount and composition of its essential amino acids [26]. Macroalgae are an important source of proteins because their protein content is rich in essential amino acids (histidine, isoleucine, leucine, lysine, methionine, phenylalanine, threonine, tryptophan and valine) [68]. In this regard, macroalgae proteins are also significant as a source of peptides and amino acid extracts, principally after enzymatic digestion, which increases their solubility in water, making them acceptable to be employed in a variety of industries [69].

Red macroalgae *Palmaria palmata* exhibited a similar pattern, displaying variations in macroalgal protein content, with the winter–spring season showing greater protein content than the summer–early autumn season [70]. Protein content differs greatly with seasons; the highest concentration was recorded during the beginning of spring and winter, while the lowest concentration was recorded in the early autumn and summer season [71]. Afonso et al. [72] proposed that a gradual decrease in protein levels from March to August may be due to the lower availability of nitrogenous compounds. However, other seasonal factors may also influence the protein content, namely the high temperature of water, salinity and eutrophication [71,72]. Balboa et al. [51] indicated that protein content exhibited a negative relationship with temperature and salinity. Overall, the macroalgal protein is considered an excellent source of EAAs and represents almost half of the total AAs they produce [73].

#### 3.3.3. Lipids and Fatty Acids

Lipids play a basic role not only in energy supply, but they are also necessary for the production of hormones and for maintaining the integrity of cell membranes [74]. Lipids are also required for the transportation and absorption of fat-soluble vitamins, including A, D, E and K [75]. Therefore, a total of 16 individual FAs were identified and quantified in the five macroalgal species studied, including 8 saturated fatty acids (SFAs), 6 monounsaturated fatty acids (MUFAs) and 2 polyunsaturated fatty acids (PUFAs). Palmitic (C16:0) and stearic (C18:0) acids were the most abundant SFA in all macroalgal species, with high concentrations observed in the summer season (significant difference at *p* < 0.05). The greatest content of MUFAs was recorded for oleic acid (C18:1) in the summer season for all macroalgae studied, except *T. ornata* in the winter season. Oleic acid (C18:1) was followed by eicosenoic (C20:1), heptadecenoic (C17:1), palmitoleic (C16:1), and finally, tetracosenoic (C24:1) acid, at a lower concentration. Two PUFA were reported, namely linoleic (C18:2 ω-6) and linolenic (C18:3 ω-3) acid, with slightly higher concentrations in the summer than in the winter season in *C. prolifera* and *T. ornata*, while there was no difference in their amounts in both seasons in *C. myrica, C. trinodis* and *A. spicifera* (Table 5).

Oleic acid (C18:1) followed by palmitic acid (C16:0) were the most abundant FA, in agreement with Morales et al. [76]. PUFA help macroalgae survive by acting as precursors for the biosynthesis of a variety of secondary metabolites with crucial ecological roles [77]. Khairy and El shafey [64] reported that palmitic acid (C16:0) is the most abundant saturated fatty acid in seaweeds, accounting for 74.3%. The essential C18 fatty acids, linoleic acid (18:2, ω6) and linolenic acid (18:3, ω3), were recorded in the same amounts, with the highest contents in March and April (5.7–7.2%) [51]. Macroalgal lipid contents are directly affected by many variables, such as macroalgal species, location, sampling period and environmental conditions, in addition to the extraction method and solvent polarity [78].

Although many studies proved that macroalgae possess relatively low lipid contents, their PUFAs contents are equal to or may be greater than those of terrestrial plants [59]. Macroalgae accumulate high concentrations of PUFAs, which have beneficial impacts on human health, such as reducing cardiovascular risk and improving both the brain function and immune response [32,79]. It was also described that the PUFAs content of *Caulerpa* is greater than those in coconut and palm oils [80]. In addition, Ajayan et al. [60] stated that linolenic acid and oleic acid comprised the majority of the total fatty acids of macroalgae.

Francavilla et al. [81] described the increase in PUFAs and decrease in SFAs in macroalgae *G. gracilis* during the winter season. They attributed this result to the increased tightness of cell membranes due to lower temperatures. Due to the mild winters recorded along the Egyptian coast, the lowest temperatures recorded do not seem to significantly change the PUFAs content. Balboa et al. [51] concluded that the unsaturation degree of FAs depends primarily on the water temperature; macroalgae harvested from cold water have a greater content of PUFAs and unsaturation degree than those collected from tropical water. Some seaweed fatty acids are distinctive and play crucial roles in nutrition and cell membrane construction, such as the essential α-linolenic fatty acid, which cannot be synthesized by mammals, while it can only be synthesized in limited amounts by terrestrial plants [82]. Both FAs content and profile differ based on the variation of geographical location, biotic (temperature, salinity, pH, light, nutrient) and abiotic parameters (herbivory), in addition to the genetic characteristics of each macroalgal species [51,75]. Based on the results, high FAs content can best be obtained during the summer season. SFAs, C14:0 and C16:0, are essential for the cholesterol synthesis and thus important for human health [72].

#### 3.3.4. Organic Acids

**Figure 5:**
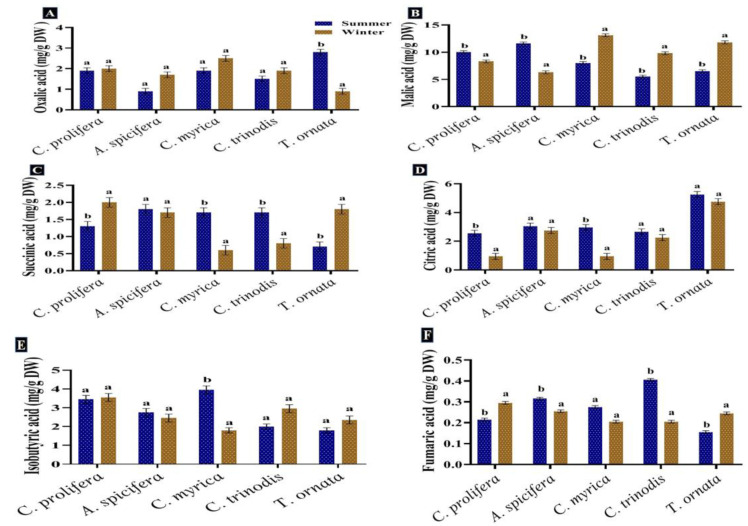
Seasonal variations in the concentrations (mg/g DW) of (**A**) Oxalic acid, (**B**) Malic acid, (**C**) Succinic acid, (**D**) Citric acid, (**E**) Isobutyric acid and (**F**) Fumaric acid in macroalgae *Caulerpa prolifera, A. spicifera, Cystoseira myrica, Cystoseira trinodis* and *T. ornata*. Values are shown as means ± S.E. (*n* = 3). Different letters show significance between the two seasons of the same species (*p* < 0.05).

Six organic acids were identified and measured in the macroalgae tested. Malic acid, isobutyric acid, citric acid and oxalic acid were the most abundant in the two seasons. The malic acid quantity showed a comparative increase in the summer season in *C. prolifera* and *A. spicifera,* but the three brown macroalgae recorded a high content in the winter. Succinic acid was found in high concentration in *A. spicifera* during the summer season, while fumaric acid was observed in minimum quantity in all the macroalgae studied (Figure 5). Carpena et al. [83] also recorded the presence of several organic acids, including malic, oxalic and citric acids, in the three seaweeds *Chondrus crispus*, *Mastocarpus stellatus* and *Gigartina pistillata*. Tanna et al. [82] also reported that lactic and oxalic acids were found in the green macroalgae *Caulerpa scalpelliformis*.

These detected organic acids are known for their high biological and medical values. Succinic acid has high potential in many biological production processes, including food, pharmaceutical, cosmetics, detergents and lubricants [84]. Fumaric acid is an intermediate of the TCA cycle, and it is generally used in the food industries, such as a beverage constituent and food acidulant [85]. Malic acid, as a low-calorie food additive, is used in a variety of industries, including food, beverage, metal cleaning, pharmaceuticals and plastics [86]. Malic and citric acids have antioxidant properties and are frequently used in the food, agriculture, pharmaceutical and chemical industries [83]. Butyric acid is used to produce butyric acid esters, cellulose butyrate, food and medicine, as well as serving as an emulsifier, varnish and cosmetic [87].

### 3.4. Secondary Metabolites

#### Phenolic Compounds

**Figure 6:**
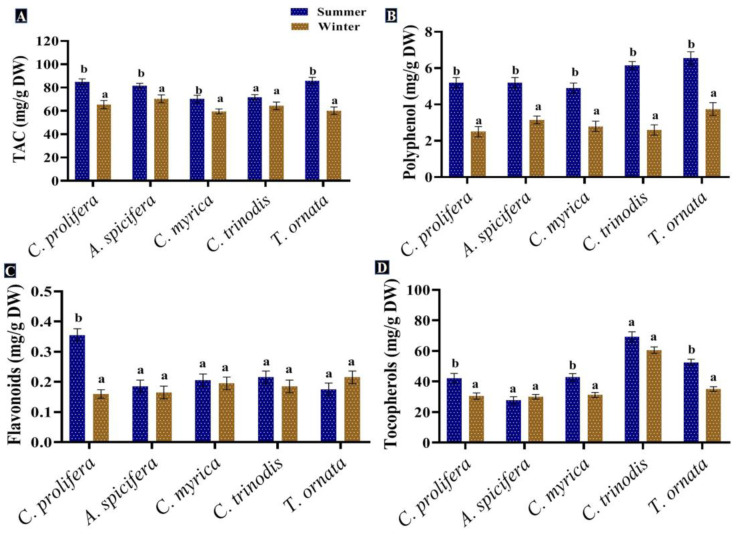
Seasonal variations in the concentrations (mg/g DW) of (**A**) TAC, (**B**) Polyphenol, (**C**) Flavonoids and (**D**) Tocopherols in macroalgae *Caulerpa prolifera, A. spicifera, Cystoseira myrica, Cystoseira trinodis* and *T. ornata*. Values are shown as means ± S.E. (*n* = 3). Different letters show significance between the two seasons of the same species (*p* < 0.05).

Phenolic compounds are a group of metabolites with the most structural variety and the greatest concentration in macroalgae [88]. Phenolic compounds produced by seaweeds in the present study were assessed and quantified in the five macroalgal species tested during the two seasons (summer and winter). The greatest levels of phenolic compounds, such as polyphenols and flavonoids, were recorded in the summer season for all macroalgal species studied. In contrast, tocopherols recorded a slight increase in the winter (31.1 mg/g DW) compared to the summer season (29.4 mg/g DW) (Figure 6).

The total phenolic content of macroalgae changes with seasonal variations in temperature, salinity, light intensity, geographical region and water depth, in addition to other biological factors, such as age, size, the stage of the seaweed’s life cycle and herbivores’ presence [89]. The greatest levels of phenolic compounds were found in the summer season. Schiener et al. [55] concluded that the highest polyphenol quantity was observed between May and July in all seaweeds tested, while the lowest quantity was found in October for the *Laminaria* spp. and March for the *Alaria esculenta* and *Saccharina latissima*. Mancuso et al. [90] proved an increase in the total phenolic content in brown seaweed *Cystoseira compressa* as the water temperature rose. This may be attributed to greater light irradiance during the spring season; the exposure of seaweeds to UV radiation promotes the formation of phenolic compounds to provide protection from oxidative stress [91]. Polyphenolic compounds extracted from macroalgae exhibited antioxidant [92], anti-inflammatory and antidiabetic [93], anticarcinogenic [94] and antimicrobial properties [7]. Moreover, these compounds could be used in several industries and applications, generating innovative products, such as natural food stabilizer, skin care and anti-aging cosmetic products [32,88].

There is variability in seaweeds’ phenolic content throughout the year, which represents the cellular defensive response, as well as prevents the attack of bacteria, microalgae, fungi, invertebrates, and enables survival in these difficult conditions [95]. The strong antioxidant potential of macroalgae was attributed to the higher levels of antioxidant molecules, such as flavonoids, ascorbate, phenols and glutathione. Wang et al. [96] reported that seaweed phenols have scavenging potential because of the presence of a hydroxyl group, which is a remarkable constituent of seaweed.

In general, flavonoids are found in epidermal cells to absorb UV light; therefore, their concentration is higher in the summer season, with high light intensity and duration [97]. Water salinity decreases during the rainy season, lower salinity effects the biochemical composition of macroalgae by reducing their phenolic content [98]. Marinho et al. [99] recorded the same seasonal fluctuation pattern of flavonoids’ concentration in *Saccharina latissima*. Seasonal environmental factors may result in a considerable difference in antioxidant activity [97]. The total antioxidant activity (TAC) was enhanced in the summer compared to the winter season in all studied macroalgal species.

## 4. Conclusions

Out of several macroalgae, *Caulerpa prolifera* (green macroalgae), *Acanthophora spicifera* (red macroalgae) and *Cystoseira myrica*, *Cystoseira trinodis* and *Turbinaria ornata* (brown macroalgae) were the most dominant species in the three target collection sites along the Red Sea, Egypt. Macroalgae identification was confirmed using the molecular (18s rRNA) approach. The majority of primary and secondary metabolites, as well as total antioxidant activity, were enhanced in the summer season. The present study revealed significant seasonal and species variations in the biochemical composition of the macroalgal species collected and reported that the macroalgae under study possess greater nutritional value in the summer compared to the winter season.

## Figures and Tables

**Table 1:** Physico-chemical analysis of water samples collected from macroalgae collection sites.

Water Analysis	Site 1		Site 2		Site 3	
	Season					
	Summer	Winter	Summer	Winter	Summer	Winter
Temperature (°C)	31.5 ± 2	18.5 ± 2	32.7 ± 2	17.5 ± 2	32.2 ± 2	15.8 ± 2
PH	7.89 ± 0.2	7.78 ± 0.1	7.82 ± 0.2	7.76 ± 0.2	7.8 ± 0.2	7.72 ± 0.1
Total alkalinity	113.3 ± 5.1	127.4 ± 6.3	126.6 ± 5.1	125.4 ± 5.2	116.6 ± 3.9	125.4 ± 4.2
Nitrates (ppm)	43.44 ± 2.1	109.8 ± 4.5	42.85 ± 2.3	98.17 ± 4.1	21.70 ± 1.5	33.11 ± 2.2
Chlorides (ppm)	18,345 ± 120	1918 ± 55	17,281 ± 118	19,409 ± 106	19,159 ± 124	20,486 ± 154
Sulphate (ppm)	2767 ± 53	2913 ± 48	2489 ± 38	2953 ± 54	2859 ± 51	3148 ± 62
Phosphate (ppm)	0.55 ± 0.02	NR	1.99 ±0.05	ND	ND	0.59 ±0.03
Sodium (ppm)	11,261 ± 75	13,474 ± 83	9932 ± 65	13,029 ± 79	13,576 ± 102	12,519 ± 98
Potassium (ppm)	506 ± 19	558 ± 25	479 ± 22	617 ± 29	571 ± 18	601 ± 21
Calcium (ppm)	454 ± 17	476 ± 22	438 ± 25	508 ± 19	433 ± 22	529 ± 23
Magnesium (ppm)	1327 ± 32	1645 ± 38	1214 ± 28	1432 ± 39	1384 ± 35	1438 ± 35
Iron (ppm)	NR	ND	0.43 ± 0.02	ND	ND	ND
Copper (ppm)	NR	ND	NR	NR	ND	ND
Manganese (ppm)	0.027 ± 0.001	0.032 ± 0.002	0.036 ± 0.002	0.024 ± 0.001	0.029 ± 0.002	0.026 ± 0.003
Phosphorus (ppm)	0.18 ± 0.02	NR	0.65 ± 0.01	ND	ND	0.195 ± 0.02
Carbonate (ppm)	41.7 ± 4	40.8 ± 3	38.5 ± 4	41.2 ± 3	38.5 ± 2	40.4 ± 3

**Table 2:** Polymerase chain reaction primers used in the present study.

Sample ID	Barcode Sequence	Linker Primer Sequence
A1	“GCCACATA, GGTGCGAA”	“GCGGTAATTCCAGCTCCAA,AATCCRAGAATTTCACCTCT”
A2	“GCCACATA, GTCGTAGA”	“GCGGTAATTCCAGCTCCAA,AATCCRAGAATTTCACCTCT”
A3	“GCCACATA, TCTTCACA”	“GCGGTAATTCCAGCTCCAA,AATCCRAGAATTTCACCTCT”
A4	“GCCACATA, TTCACGCA”	“GCGGTAATTCCAGCTCCAA,AATCCRAGAATTTCACCTCT”
A5	“GCCACATA, AATCCGTC”	“GCGGTAATTCCAGCTCCAA,AATCCRAGAATTTCACCTCT”

**Table 3:** Seasonal variations in the concentrations (mg/g DW) of minerals in macroalgae *C. prolifera, A. spicifera, C. myrica, C. trinodis* and *T. ornata*. Values are shown as means ± S.E. (*n* = 3). Different letters show significance between the two seasons of the same species (*p* < 0.05).

Minerals	*C. prolifera*		*A. spicifera*		*C. myrica*		*C. trinodis*		*T. ornata*	
	Summer	Winter	Summer	Winter	Summer	Winter	Summer	Winter	Summer	Winter
P	1.6 ± 0.03 b	2.67 ± 0.15 a	3.53 ± 0.21 a	3.76 ± 0.36 a	5.62 ± 0.18 b	3.44 ± 0.16 a	4.25 ± 0.29 b	2.61 ± 0.31 a	3.1 ± 0.18 b	4.2 ± 0.34 a
K	0.64 ± 0.03 b	1.42 ± 0.07 a	1.96 ± 0.02 a	1.27 ± 0.20 a	1.89 ± 0.06 b	0.94 ± 0.04 a	2.18 ± 0.04 a	1.95 ± 0.31 a	1.29 ± 0.13 b	2.54 ± 0 a
Na	0.29 ± 0 b	0.37 ± 0.01 a	0.57 ± 0.05 b	0.8 ± 0.08 a	0.81 ± 0.02 b	0.63 ± 0.03 a	0.91 ± 0.06 b	0.6 ± 0.03 a	0.4 ± 0.09 b	0.84 ± 0.06 a
Mg	0.23 ± 0.02 b	0.41 ± 0.01 a	0.53 ± 0.04 a	0.57 ± 0.09 a	0.82 ± 0.02 b	0.49 ± 0.02 a	0.8 ± 0.06 b	0.54 ± 0.06 a	0.52 ± 0.10 b	0.74 ± 0.08 a
Ca	0.02 ± 0 a	0.03 ± 0 a	0.04 ± 0 a	0.03 ± 0.01 a	0.04 ± 0 a	0.03 ± 0 a	0.05 ± 0 a	0.04 ± 0 a	0.04 ± 0.01 b	0.06 ± 0 a
Cd	0.21 ± 0.01 b	0.03 ± 0.01 a	0.21 ± 0.01 a	0.17 ± 0 a	0.16 ± 0.01 b	0.13 ± 0 a	0.2 ± 0.01 b	0.08 ± 0.01 a	0.23 ± 0.01 b	0.06 ± 0.01 a
Fe	0.04 ± 0 b	0.06 ± 0.01 a	0.07 ± 0.01 a	0.07 ± 0.01 a	0.1 ± 0 a	0.07 ± 0 a	0.1 ± 0.01 b	0.06 ± 0 a	0.07 ± 0.01 a	0.1 ± 0.01 a
Zn	0.03 ± 0 b	0.06 ± 0.01 a	0.08 ± 0.01 a	0.08 ± 0.01 a	0.11 ± 0 b	0.08 ± 0 a	0.1 ± 0.02 b	0.08 ± 0 a	0.1 ± 0.01 a	0.09 ± 0 a
Mn	0.02 ± 0 a	0.03 ± 0 a	0.04 ± 0 a	0.05 ± 0.01 a	0.07 ± 0 b	0.05 ± 0 a	0.05 ± 0 a	0.04 ± 0 a	0.04 ± 0.01 a	0.05 ± 0 a
Cu	0.01 ± 0 a	0.01 ± 0 a	0.01 ± 0.01 b	0.02 ± 0 a	0.02 ± 0 a	0.02 ± 0 a	0.02 ± 0.001 a	0.01 ± 0 a	0.02 ± 0 a	0.02 ± 0 a

**Table 4:** Seasonal variations in the concentrations (mg/g DW) of amino acids—essential (EAAs) and non-essential amino acids (NEAAs)—in macroalgae *C. prolifera, A. spicifera, C. myrica, C. trinodis* and *T. ornata*. Values are shown as means ± S.E. (*n* = 3). Different letters show significance between the two seasons of the same species (*p* < 0.05).

Amino Acids	*C. prolifera*		*A. spicifera*		*C. myrica*		*C. trinodis*		*T. ornata*	
	Summer	Winter	Summer	Winter	Summer	Winter	Summer	Winter	Summer	Winter
EAAs										
Lysine	3.6 ± 0.30 a	3.2 ± 0.10 a	2.8 ± 0.20 a	2.6 ± 0.10 a	3.1 ± 0.20 b	6.2 ± 0.30 a	3.7 ± 0.30 a	3.9 ± 0.10 a	3.1 ± 0.20 a	2.1 ± 0.10 a
Histidine	1.1 ± 0.10 a	1.2 ± 0 a	1.1 ± 0.10 a	1 ± 0.20 a	1.1 ± 0.10 a	1.1 ± 0.20 a	1.5 ± 0.20 a	1.4 ± 0.10 a	0.9 ± 0.10 b	1.4 ± 0.10 a
Phenylalanine	1.01 ± 0.10 b	0.53 ± 0.01 a	1.19 ± 0.08 b	0.34 ± 0.08 a	0.52 ± 0.09 b	0.27 ± 0.09 a	1.31 ± 0.11 b	0.91 ± 0.13 a	1.14 ± 0.08 b	0.67 ± 0.13 a
Valine	0.91 ± 0.03 b	0.34 ± 0.01 a	0.73 ± 0.04 a	0.72 ± 0.02 a	0.95 ± 0.03 b	2.22 ± 0.03 a	0.71 ± 0.05 b	0.63 ± 0.02 a	0.62 ± 0.04 b	0.38 ± 0.01 a
Threonine	0.29 ± 0.12 b	0.15 ± 0.04 a	0.63 ± 0.08 b	0.31 ± 0.10 a	0.35 ± 0.12 a	0.38 ± 0.33 a	0.55 ± 0.08 b	0.21 ± 0.08 a	0.29 ± 0.07 a	0.57 ± 0.04 a
Isoleucine	0.11 ± 0 a	0.09 ± 0 a	0.19 ± 0.03 a	0.16 ± 0 a	0.13 ± 0.01 a	0.16 ± 0 a	0.15 ± 0.02 a	0.17 ± 0.01 a	0.12 ± 0.01 b	1.77 ± 0.02 a
Methionine	0.03 ± 0.01 a	0.03 ± 0.01 a	0.3 ± 0.06 b	0.05 ± 0.02 a	0.13 ± 0.01 b	0.03 ± 0 a	0.24 ± 0.05 b	0.07 ± 0.01 a	0.11 ± 0.02 b	0.31 ± 0.06 a
Leucine	0.02 ± 0 a	0.02 ± 0 a	0.26 ± 0.02 a	0.02 ± 0 a	0.15 ± 0.01 b	0.03 ± 0 a	0.19 ± 0.02 b	0.08 ± 0.01 a	0.1 ± 0.01 b	0.24 ± 0.02 a
**ΣEAAs**	7.07	5.56	7.20	5.20	6.43	10.39	8.35	7.37	6.38	7.44
NEAAs										
Glycine	79.5 ± 8 b	57.1 ± 3 a	49.5 ± 4.9 b	37.7 ± 1.9 a	57.8 ± 5.7 a	41.6 ± 1.8 a	40.1 ± 3.8 b	70.9 ± 3.8 a	49.8 ± 4.9 b	34 ± 1.8 a
Alanine	19.6 ± 2 b	4.2 ± 0.2 a	4.5 ± 0.4 b	11.2 ± 0.5 a	8.5 ± 0.8 a	9.1 ± 0.4 a	7.6 ± 0.7 b	12.4 ± 0.6 a	11.8 ± 1.2 b	5.8 ± 0.2 a
Asparagine	0.83 ± 0.07 b	0.63 ± 0.03 a	4.49 ± 0.4 b	1.17 ± 0.05 a	1.2 ± 0.1 b	0.31 ± 0.02 a	3.9 ± 0.4 b	0.58 ± 0.02 a	1.37 ± 0.1 b	5.04 ± 0.21 a
Glutamic acid	0.78 ± 0.01 b	0.09 ± 0.01 a	0.7 ± 0.01 b	0.55 ± 0.01 a	0.65 ± 0.01 a	0.65 ± 0.01 a	0.93 ± 0.02 a	0.98 ± 0.01 a	0.67 ± 0.01 b	0.92 ± 0.01 a
Arginine	0.7 ± 0.08 b	0.5 ± 0.03 a	0.8 ± 0.06 a	0.8 ± 0.18 a	0.8 ± 0.07 b	1.4 ± 0.06 a	1.4 ± 0.12 b	1 ± 0.04 a	1.3 ± 0.39 a	1.1 ± 0.04 a
Glutamine	0.63 ± 0.1 a	0.63 ± 0.08 a	1.79 ± 0.62 a	1.95 ± 0.13 a	1.16 ± 0.14 a	1.13 ± 0.04 a	2.77 ± 0.4 b	0.71 ± 0.07 a	1.39 ± 0.1 b	2.05 ± 0.69 a
Serine	0.54 ± 0.1 b	0.28 ± 0.08 a	0.65 ± 0.14 b	0.24 ± 0.02 a	0.42 ± 0.04 b	0.21 ± 0.01 a	0.76 ± 0.14 b	0.38 ± 0.08 a	0.63 ± 0.13 b	0.22 ± 0.05 a
Tyrosine	0.47 ± 0.05 a	0.7 ± 0.04 a	0.59 ± 0.06 b	0.37 ± 0.02 a	0.36 ± 0.04 b	0.76 ± 0.05 a	0.46 ± 0.05 b	0.89 ± 0.05 a	0.07 ± 0.01 b	0.7 ± 0.04 a
Ornithine	0.28 ± 0.02 b	0.13 ± 0.05 a	0.34 ± 0.03 b	0.21 ± 0.02 a	0.32 ± 0.04 b	0.24 ± 0.02 a	0.59 ± 0.11 b	0.34 ± 0.02 a	0.67 ± 0.1 a	0.55 ± 0.29 a
Aspartate	0.15 ± 0 b	0.21 ± 0.08 a	0.16 ± 0 b	0.08 ± 0 a	0.09 ± 0 a	0.07 ± 0 a	0.19 ± 0 a	0.15 ± 0 a	0.15 ± 0 a	0.19 ± 0.02 a
Cysteine	0.11 ± 0.06 b	0.73 ± 0.07 a	0.12 ± 0.08 a	0.12 ± 0.05 a	0.08 ± 0.05 a	0.05 ± 0.11 a	0.15 ± 0.06 b	0.07 ± 0.13 a	0.02 ± 0.01 b	0.39 ± 0.1 a
**ΣNEAAs**	103.59	65.20	63.64	54.39	71.38	55.52	58.85	88.40	67.87	50.96

**Table 5:** Seasonal variations in the concentrations (mg/g DW) of various fatty acids in macroalgae *C. prolifera, A. spicifera, C. myrica, C. trinodis* and *T. ornata*. Saturated fatty acids (SFAs), monounsaturated fatty acids (MUFAs), polyunsaturated fatty acids (PUFAs). Values are shown as means ± S.E. (*n* = 3). Different letters show significance between the two seasons of the same species (*p* < 0.05).

Fatty Acids	*C. prolifera*		*A. spicifera*		*C. myrica*		*C. trinodis*		*T. ornata*	
	Summer	Winter	Summer	Winter	Summer	Winter	Summer	Winter	Summer	Winter
SFAs										
Palmitic (C16:0)	34.7 ± 17 a	25.1 ± 10 a	33.7 ± 8.5 b	21.1 ± 5.8 a	35.4 ± 10 b	23.5 ± 6 a	27.3 ± 6 a	26.3 ± 13 a	29 ± 9.4 b	22.7 ± 4 a
Myristic (C14:0)	3.7 ± 20.9 b	2.4 ± 13 a	3.1 ± 12.9 b	1.9 ± 8.5 a	3.6 ± 15.1 a	2.7 ± 9.1 a	3.4 ± 10.3 a	2.8 ± 16.1 a	2.9 ± 13 b	1.9 ± 7.7 a
Stearic (C18:0)	1.7 ± 0.2 a	1.7 ± 0.1 a	2.6 ± 0.1 b	1.6 ± 0.1 a	1.5 ± 0.1 a	1.2 ± 0.1 a	2.6 ± 0.1 b	1.8 ± 0.1 a	1.7 ± 0.1 a	1.3 ± 0.1 a
Arachidic (C20:0)	1.1 ± 0.11 a	1.3 ± 0.02 a	1.6 ± 0.15 a	1.1 ± 0.02 a	0.8 ± 0.02 a	1 ± 0.03 a	1.8 ± 0.05 a	1.1 ± 0.04 a	1.1 ± 0.02 a	1.5 ± 0.15 a
Docosanoic (C22:0)	0.7 ± 0.04 a	0.6 ± 0.03 a	0.5 ± 0.06 a	0.5 ± 0.02 a	0.6 ± 0.02 b	0.4 ± 0.02 a	0.6 ± 0.09 a	0.5 ± 0.03 a	0.4 ± 0.02 b	0.7 ± 0.09 a
Pentacosanoic (C25:0)	0.16 ± 21 b	0.08 ± 13.1 a	0.08 ± 13 a	0.07 ± 8.6 a	0.1 ± 15.2 a	0.07 ± 9.2 a	0.11 ± 10.4 a	0.11 ± 16.2 a	0.13 ± 13.1 b	0.09 ± 7.8 a
Heptadecanoic (C17:0)	0.07 ± 0.01 b	0.03 ± 0 a	0.05 ± 0 b	0.03 ± 0 a	0.04 ± 0 a	0.03 ± 0 a	0.03 ± 0 a	0.04 ± 0 a	0.07 ± 0 b	0.05 ± 0 a
Tricosanoic (C23:0)	0.04 ± 0.01 b	0.02 ± 0 a	0.042 ± 0.02 b	0.02 ± 0.02 a	0.043 ± 0.04 b	0.02 ± 0.01 a	0.039 ± 0.01 b	0.02 ± 0.01 a	0.065 ± 0.02 b	0.03 ± 0.01 a
**Σ SFAs**	42.17	31.23	41.67	26.32	42.08	28.92	35.88	32.67	35.37	28.27
MUFAs										
Oleic (C18:1)	47.7 ± 1.8 b	35.9 ± 0.9 a	37.1 ± 0.4 a	37.8 ± 1.3 a	42.3 ± 0.7 b	30.1 ± 1.5 a	42.6 ± 0.6 b	35.3 ± 1 a	43.4 ± 0.7 a	47.3 ± 5 a
Eicosenoic (C20:1)	4.8 ± 0.8 b	3.3 ± 12.9 a	4.2 ± 2.8 a	3.9 ± 8.4 a	4.3 ± 0.5 a	4 ± 9 a	3.3 ± 0.3 a	3.7 ± 16.1 a	4.4 ± 0.8 b	3.3 ± 7.6 a
Heptadecenoic (C17:1)	1.5 ± 0.18 a	1 ± 0.11 a	1.5 ± 0.11 a	1.2 ± 0.07 a	1.5 ± 0.13 a	1.2 ± 0.08 a	1.1 ± 0.09 a	1.1 ± 0.14 a	1.3 ± 0.11 a	1 ± 0.07 a
Palmitoleic (C16:1)	0.11 ± 0 aa	0.07 ± 0.01 a	0.08 ± 0.01 aa	0.08 ± 0.01 a	0.09 ± 0 a	0.06 ± 0.01 a	0.07 ± 0.01 a	0.07 ± 0.01 a	0.08 ± 0.01 a	0.07 ± 0 a
Eicosenoic (C20:1)	0.072 ± 0 b	0.04 ± 0 a	0.087 ± 0.01 b	0.063 ± 0 a	0.191 ± 0.19 b	0.062 ± 0 a	0.075 ± 0.01 b	0.043 ± 0 a	0.071 ± 0 b	0.056 ± 0 a
Tetracosenoic (C24:1)	0.01 ± 0.001 b	0.006 ± 0 a	0.009 ± 0 b	0.008 ± 0 a	0.009 ± 0 b	0.007 ± 0 a	0.011 ± 0 b	0.006 ± 0 a	0.001 ± 0 a	0.009 ± 0 a
**Σ MUFAs**	54.69	40.32	42.98	33.05	48.39	35.43	47.10	40.22	49.25	51.60
PUFAs										
Linoleic (C18:2 ω-6)	0.3 ± 0.2 b	0.2 ± 0.1 a	0.3 ± 0.1 a	0.3 ± 0.6 a	0.3 ± 0.2 a	0.3 ± 0.2 a	0.2 ± 0.4 a	0.2 ± 0.1 a	0.3 ± 0.2 a	0.2 ± 0.08 a
Linolenic (C18:3 ω-3)	0.3 ± 21 a	0.2 ± 13.1 a	0.3 ± 0.01 a	0.2 ± 8.6 a	0.3 ± 0.2 a	0.3 ± 9.2 a	0.2 ± 0.01 a	0.2 ± 16.2 a	0.3 ± 0.1 a	0.2 ± 7.8 a
**Σ PUFAs**	0.6	0.4	0.6	0.5	0.6	0.6	0.4	0.4	0.6	0.4

## Data Availability

Not applicable.
